# Supplementary material for: Multidimensional heuristic process for high-yield production of astaxanthin and fragrance molecules in Escherichia coli
Source: Nat Commun. 2018 May 11;9:1858. doi: 10.1038/s41467-018-04211-x (PMC5948211; doi:10.1038/s41467-018-04211-x)
Supplement: Supplementary file 1 — Supplementary information [file 41467_2018_4211_MOESM1_ESM.docx]

**Multidimensional heuristic process for high-yield production of astaxanthin and fragrance molecules in *Escherichia coli***

Zhang *et al.*

**
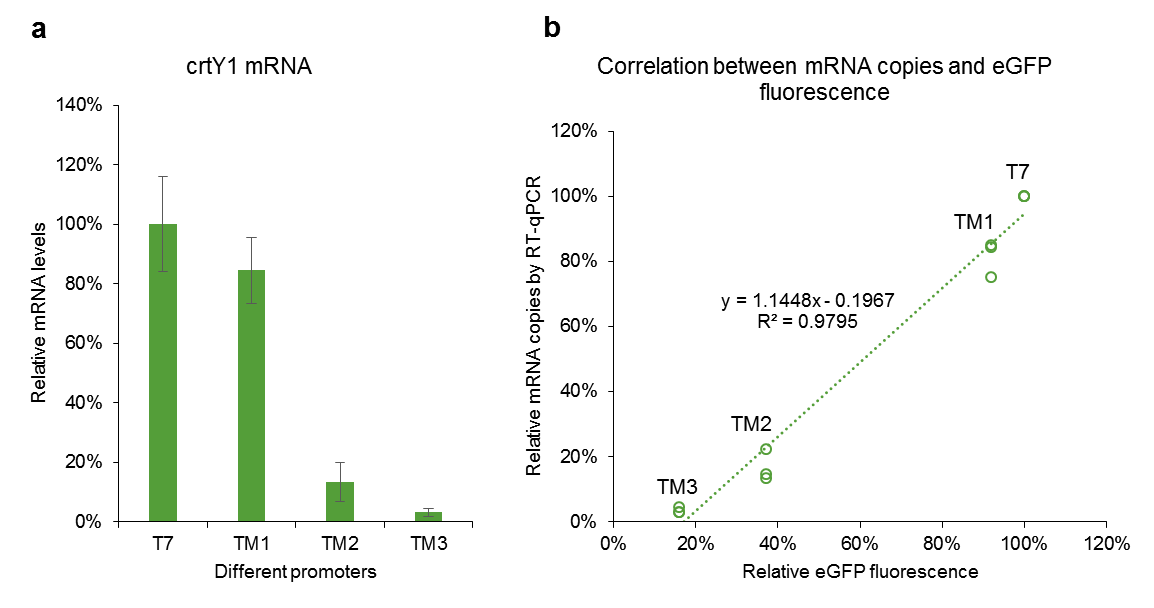
**

**Supplementary Fig. 1** Characteristics of the promoter library used in this study. (a) Quantitative real-time PCR (qPCR) measurements of *crtY1* expression and different promoters. (b) The correlation between the mRNA copies and the fluorescence of the reporter protein eGFP. The qPCR primers for measuring *crtY1* (rt-crtY1.Pa-F and rt-crtY1.Pa-R) in shown in Supplementary Table 6. Error bars, mean ± s.d., n = 3.


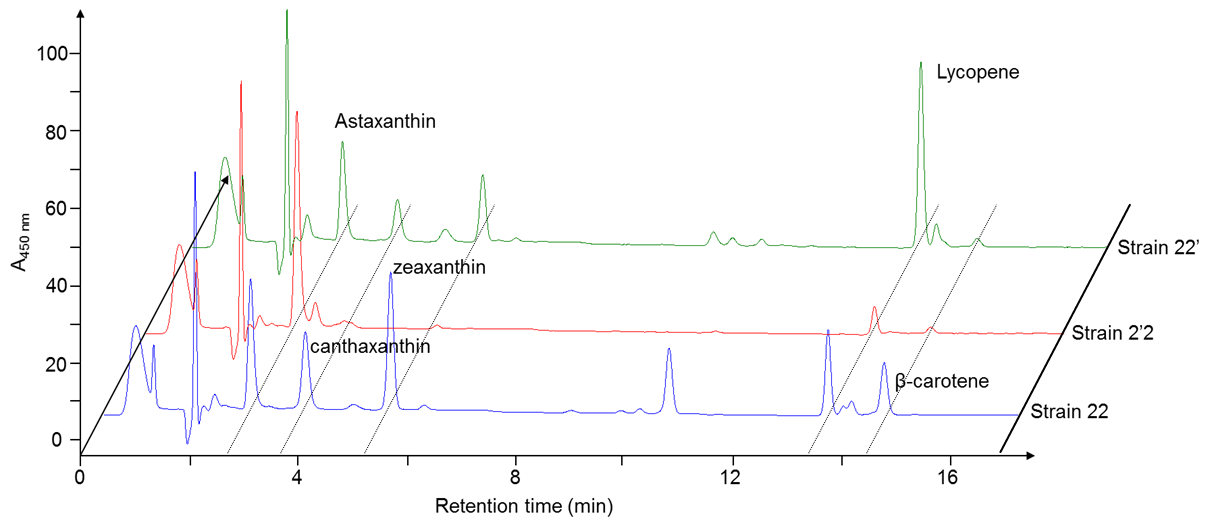


**Supplementary Fig. 2** The HPLC chromatograms of carotenoids produced in the strains 22, 2’2 and 22’ (Fig. 4b).


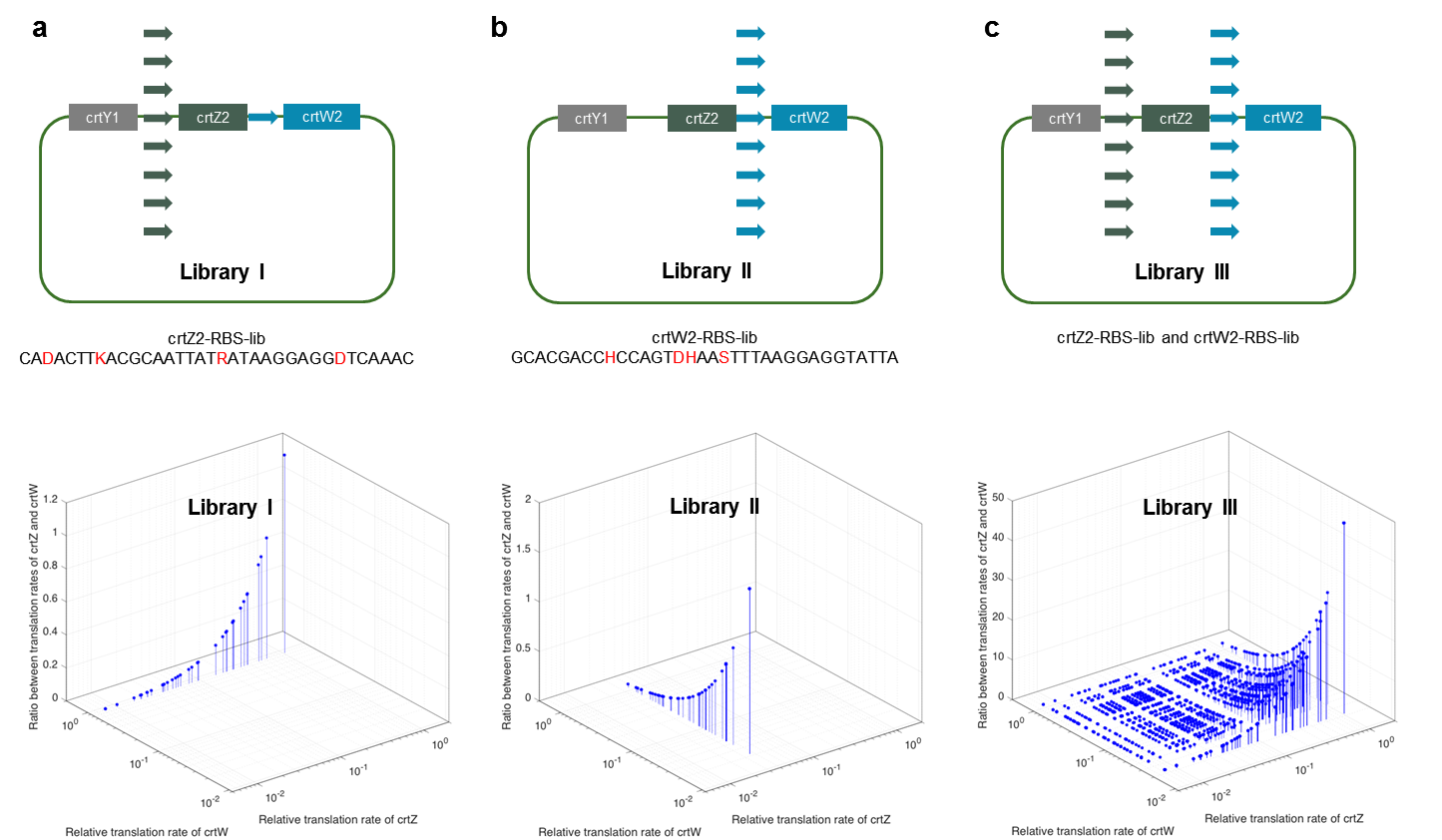


**Supplementary Fig. 3** RBS libraries used for astaxanthin optimization. (a) Library I, RBS library of *crtZ* and their predicted relative translation rates (Supplementary Table 3). (b) Library II, RBS library of *crtW* and their predicted relative translation rates (Supplementary Table 4). (c) Library III, combination of RBS library of *crtZ* and *crtW*. Nomenclature of degenerated primers is in accordance to the International Union of Pure and Applied Chemistry (IUPAC) system. The relative translation rates are calculated by normalizing to the highest translational rate for *crtZ* (80000) and *crtW* (90000).

**
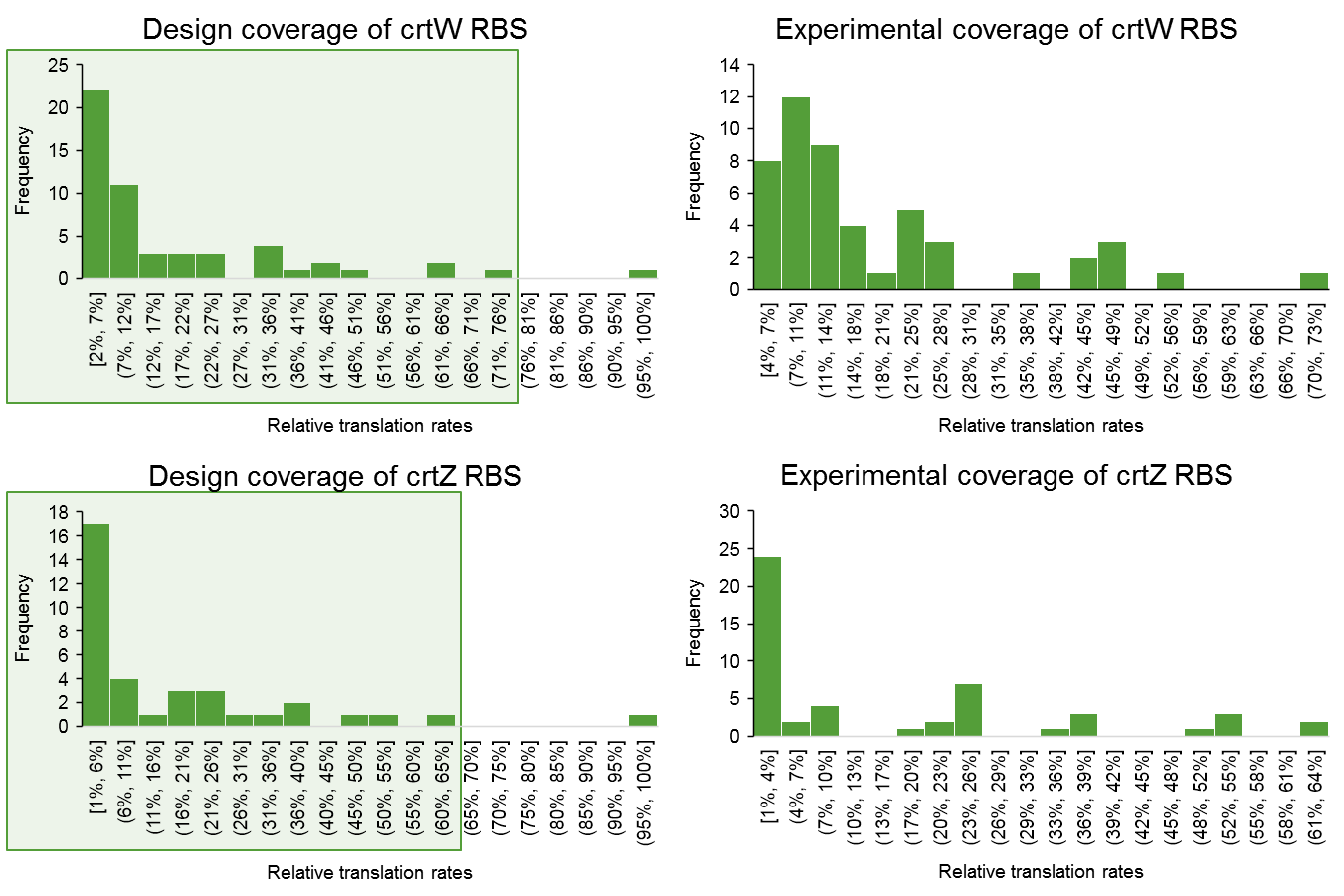
**

**Supplementary Fig. 4** Experimental validation of the RBS libraries of *crtW* and *crtZ*. Fifty colonies were chosen randomly from the Library I and II in Supplementary Fig. 2. As shown in the histogram, for both *crtW* and *crtZ* library, the fifty colonies represented ~90% of their designed RBS space. The only missing RBS is the one with the highest translational efficiency (90%~100% ranges) which has a lower probability. As in the agar plate, about 10^6^~10^8^ colonies were formed, it is likely to cover all the designed RBSs. Here, relative translational rates are calculated by normalizing to the highest translational rates for *crtZ* (80000) and *crtW* (90000).


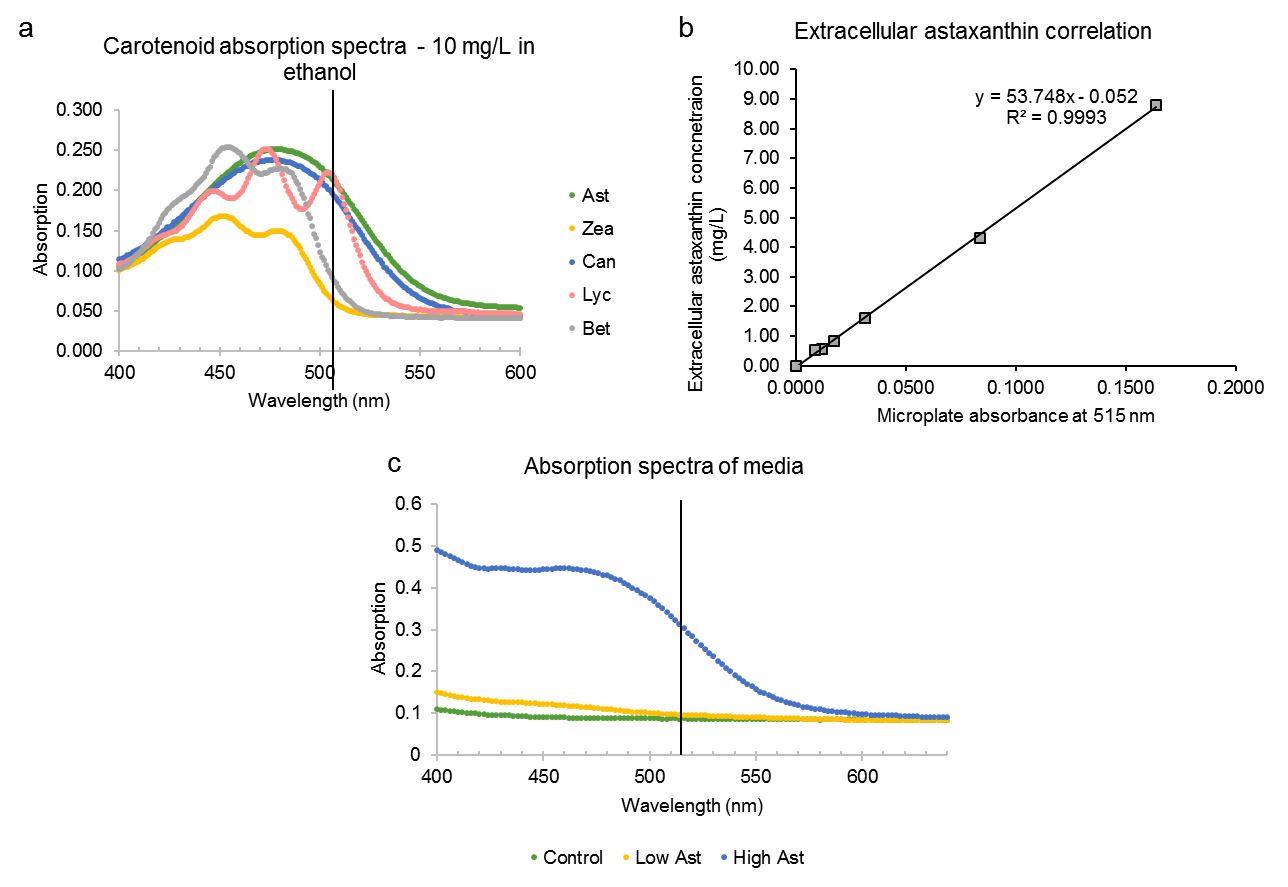


**Supplementary Fig. 5** Correlation between extracellular astaxanthin concentration and growth media absorbance. (a) Absorption spectra for the five carotenoids, Ast, astaxanthin; Zea, zeaxanthin; Can, canthaxanthin; Lyc, lycopene; Bet, β-carotene. At 515 nm, zeaxanthin and β-carotene have relatively low absorption versus the other three carotenoids. (b) A linear correlation was observed between extracellular astaxanthin concentration and medium absorbance at 515 nm. (c) The absorption spectra of different media, the ‘control’ was un-inoculated medium or background absorption, ‘Low Ast’ is the medium from strains with low astaxanthin content (strain Z2, Fig. 6), ‘High Ast’ is the medium from strains with high astaxanthin content (strain Z1, Fig. 6).


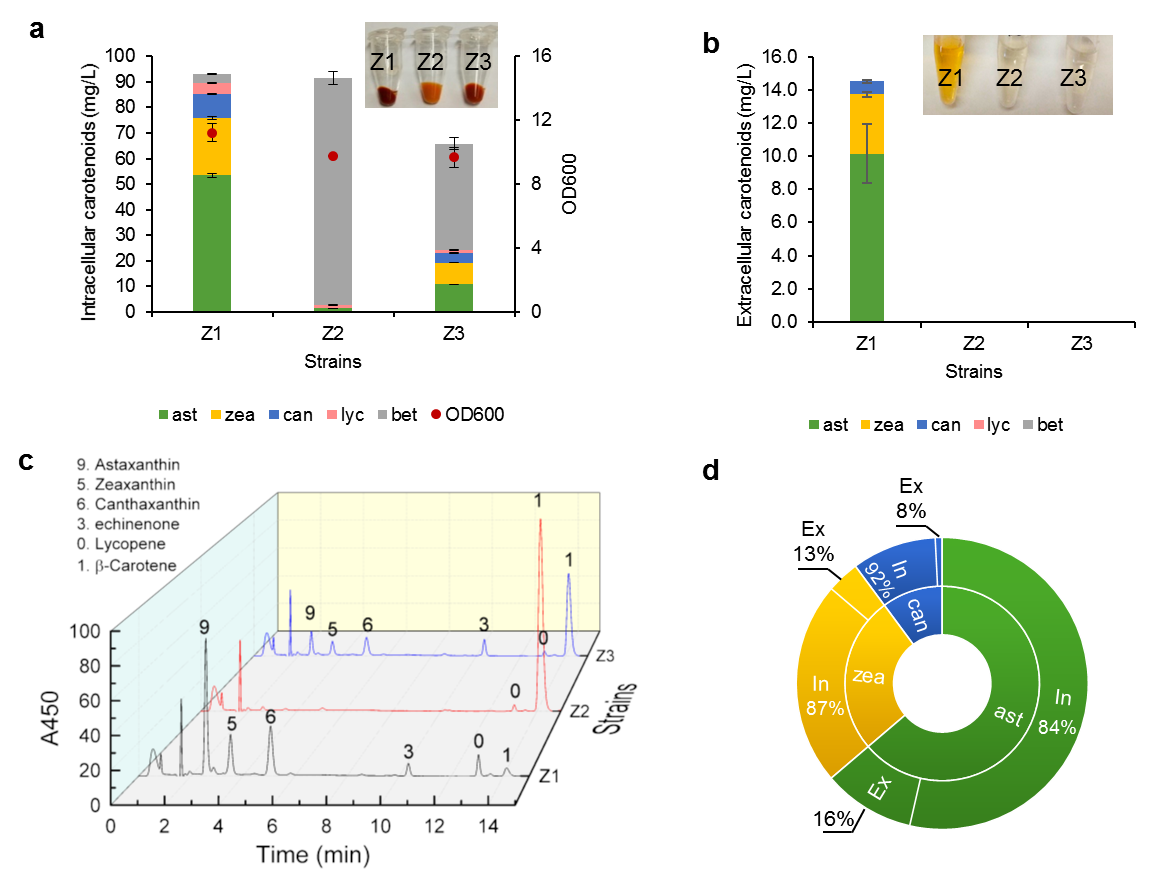


**Supplementary Fig. 6** Extracellular and intracellular carotenoids. (a) Extracellular carotenoids of strains Z1-Z3. (b) Intracellular carotenoids of strains Z1-Z3. (c) The HPLC chromatogram of carotenoids produced in the strains Z1-Z3. (d) Distribution of intra- and extra-cellular carotenoids of strain Z1. Error bars, mean ± s.d., n = 3.

**Supplementary Fig. 7** Batch and fed-batch fermentation of astaxanthin strains. The time-course profiles of OD, glucose, acetate and carotenoids production in (a) batch fermentation, (b) Fed-batch fermentation and early induction, (c) Fed-batch fermentation and late induction. The dashed arrow refers to the induction time.


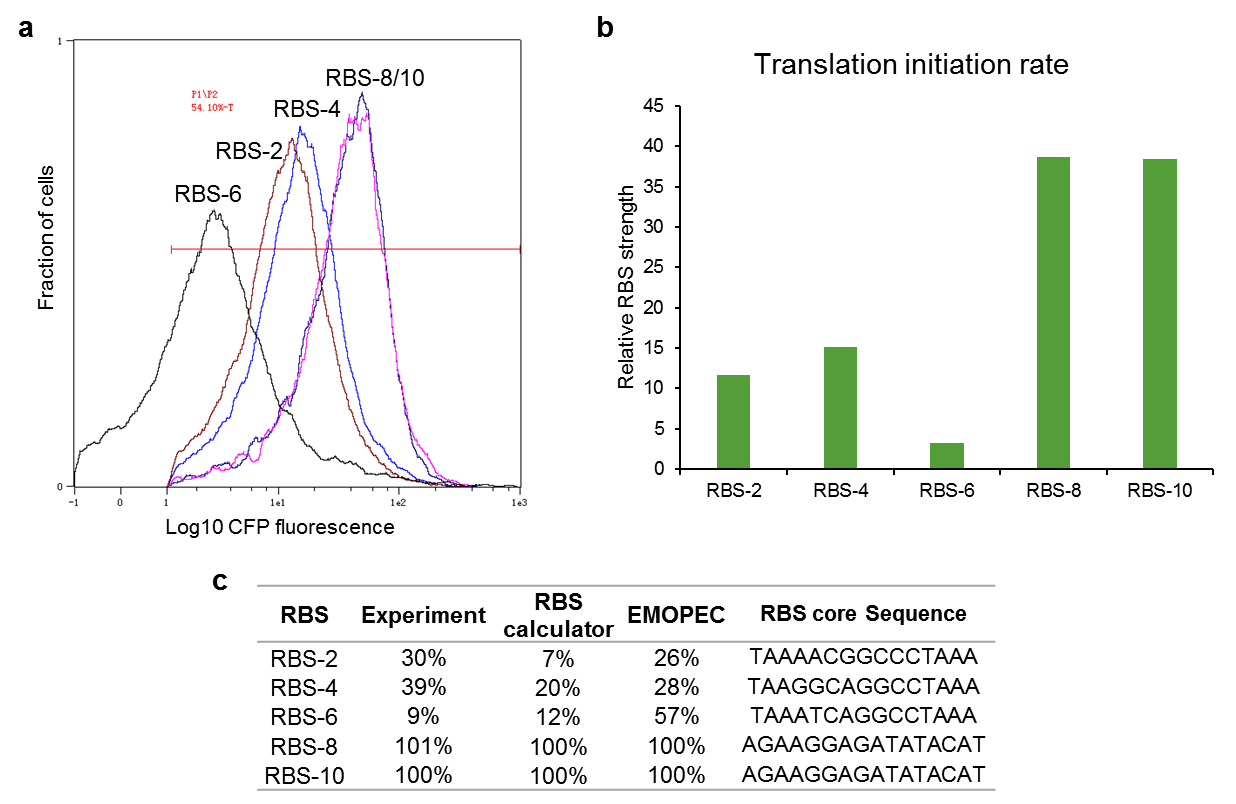


**Supplementary Fig. 8** RBS calibration using CFP. (a) Flow cytometry fluorescence measurements of cells expressing CFP (433/475 nm). (b) Relative translation initiation rates of the five RBSs. (c) Experimental values were compared with the widely-used RBS calculating algorithms, RBS calculator (version 2.0, https://salislab.net/software/) ^1^ and EMOPEC ^2^ (http://emopec.biosustain.dtu.dk/), our results indicated that both methods have certain limitations. RBS-4,6 and 8 were used in this study.


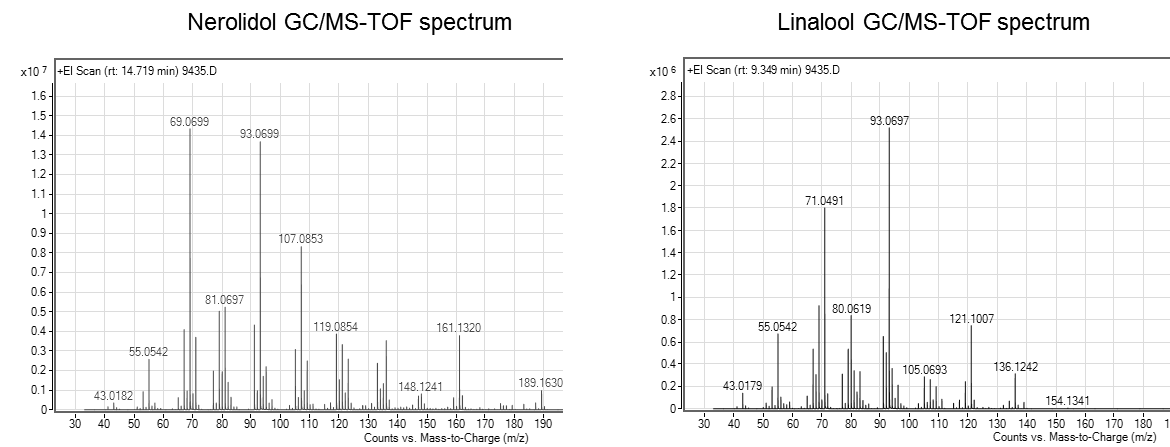


**Supplementary Fig. 9** GC/MS-TOF spectra of nerolidol and linalool.


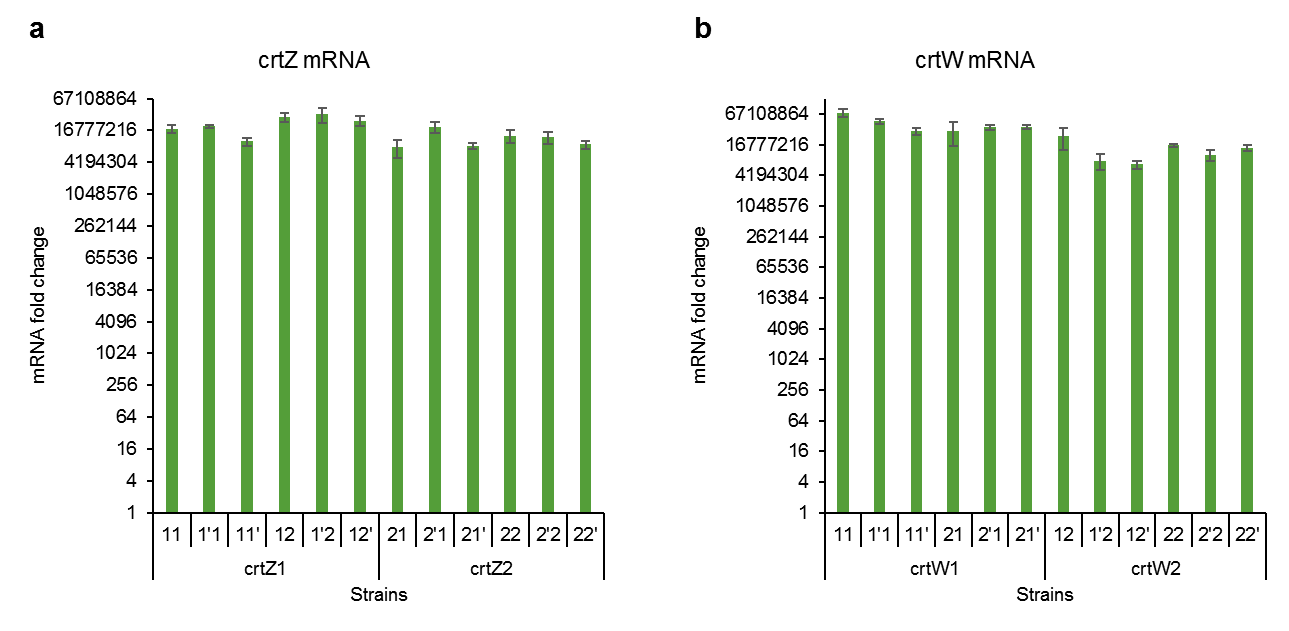


**Supplementary Fig. 10** Promoter library validation. (a) The relative mRNA levels of crtZ1 or crtZ2 in the twelve strains in Fig. 4. (b) The relative mRNA levels of crtW1 or crtW2 in the twelve strains in Fig. 4. The mRNA differences were insignificant among the twelve strains. The results were expected as all the genes were controlled by the same promoter and vector. The qPCR primers are described in **Supplementary Table 6**. Error bars, mean ± s.d., n = 6.


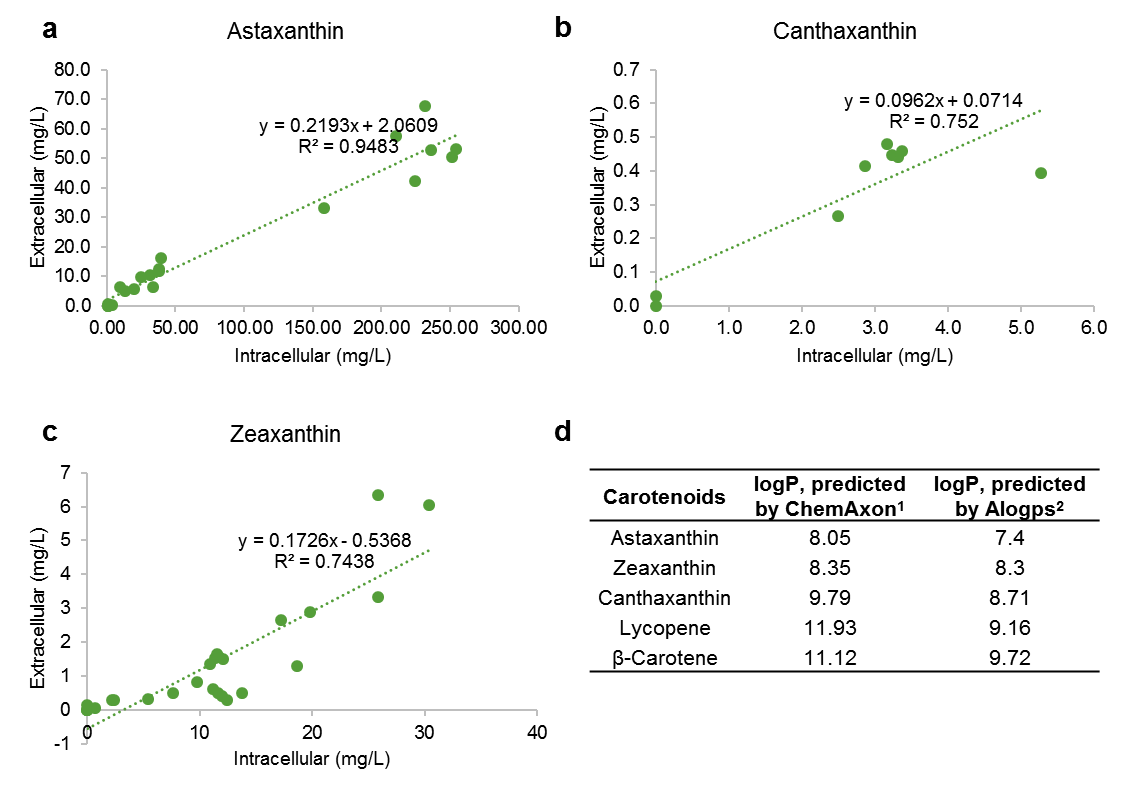


**Supplementary Fig. 11** Correlation between extracellular and intracellular carotenoids: (a) astaxanthin (including the data in flasks and bioreactors), (b) canthaxanthin, (c) zeaxanthin. (d) The octanol/water partition coefficients (logP) of the five carotenoids, calculated by ChemAxon (https://chemaxon.com/) and Alogps (<http://www.vcclab.org/lab/alogps/>)^3^. For the two most hydrophobic carotenoids, lycopene and β-carotene, their extracellular partitions were the lowest as these were undetectable in medium even though they accumulated at high concentrations intracellularly.

**
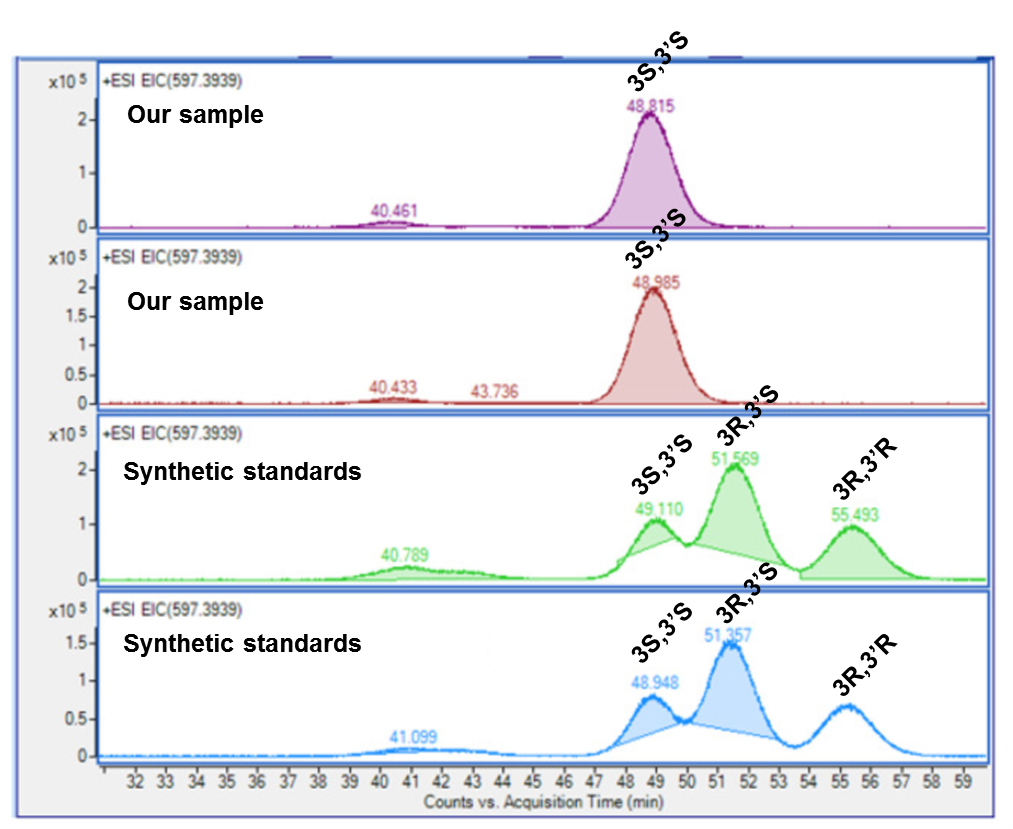
**

**Supplementary Fig. 12** Chiral analysis of astaxanthin. Our biosynthesized astaxanthin was 100% enantiopure 3S, 3′S-astaxanthin, distinct from synthetic astaxanthin that comprises a mixture of isomers of (3S, 3′S), (3R, 3′S) and (3R, 3′R).

**Supplementary Table 1**. Strain Nomenclature*

| Strain names | Global control –promoters | | | | Local control of Module 4 – RBSs and genes | | | | Products |
| --- | --- | --- | --- | --- | --- | --- | --- | --- | --- |
|  | Module 1 - SAR | Module 2 - MPPI | Module 3 - EBIA | Module 4 - YZW | RBS *crtZ* | *crtZ* | RBS *crtW* | *crtW* | / |
| Lycopene Library | T7,TM1-3 | T7, TM1-3 | T7, TM1-3 | / | / | / | / | / | Lycopene |
| 222 | TM2 | TM2 | TM2 | / | / | / | / | / | Lycopene |
| 322 | TM3 | TM2 | TM2 | / | / | / | / | / | Lycopene |
| 323 | TM3 | TM2 | TM3 | / | / | / | / | / | Lycopene |
| crtY1 | T7 | T7 | T7 | crtY1 | / | / | / |  | β-carotene |
| crtY2 | T7 | T7 | T7 | crtY2 | / | / | / | / | β-carotene |
| β-carotene Library | TM3 | TM2 | TM2 | T7, TM1-3, crtY2 | / | / | / | / | β-carotene |
| 3220 | TM3 | TM2 | TM2 | T7 | RZ1 | *crtZ*1 | RW1 | *crtW*1 | Astaxanthin |
| 3221 | TM3 | TM2 | TM2 | TM1 | RZ1 | *crtZ*1 | RW1 | *crtW*1 | Astaxanthin |
| 3222 | TM3 | TM2 | TM2 | TM2 | RZ1 | *crtZ*1 | RW1 | *crtW*1 | Astaxanthin |
| 3223 | TM3 | TM2 | TM2 | TM3 | RZ1 | *crtZ*1 | RW1 | *crtW*1 | Astaxanthin |
| 11 | TM3 | TM2 | TM2 | T7 | RZ1 | *crtZ*1 | RW1 | *crtW*1 | Astaxanthin |
| 1’1 | TM3 | TM2 | TM2 | T7 | RZ2 | *crtZ*1 | RW1 | *crtW*1 | Astaxanthin |
| 11’ | TM3 | TM2 | TM2 | T7 | RZ1 | *crtZ*1 | RW2 | *crtW*1 | Astaxanthin |
| 12 | TM3 | TM2 | TM2 | T7 | RZ1 | *crtZ*1 | RW3 | *crtW*2 | Astaxanthin |
| 1’2 | TM3 | TM2 | TM2 | T7 | RZ2 | *crtZ*1 | RW3 | *crtW*2 | Astaxanthin |
| 12’ | TM3 | TM2 | TM2 | T7 | RZ1 | *crtZ*1 | RW4 | *crtW*2 | Astaxanthin |
| 21 | TM3 | TM2 | TM2 | T7 | RZ3 | *crtZ*2 | RW1 | *crtW*1 | Astaxanthin |
| 2’1 | TM3 | TM2 | TM2 | T7 | RZ4 | *crtZ*2 | RW1 | *crtW*1 | Astaxanthin |
| 21’ | TM3 | TM2 | TM2 | T7 | RZ3 | *crtZ*2 | RW2 | *crtW*1 | Astaxanthin |
| 22 | TM3 | TM2 | TM2 | T7 | RZ3 | *crtZ*2 | RW3 | *crtW*2 | Astaxanthin |
| 2’2 | TM3 | TM2 | TM2 | T7 | RZ4 | *crtZ*2 | RW3 | *crtW*2 | Astaxanthin |
| 22’ | TM3 | TM2 | TM2 | T7 | RZ3 | *crtZ*2 | RW4 | *crtW*2 | Astaxanthin |
| Library I** | TM3 | TM2 | TM2 | T7 | RLZ1-36 | *crtZ*2 | RW3 | *crtW*2 | Astaxanthin |
| Library II** | TM3 | TM2 | TM2 | T7 | RZ3 | *crtZ*2 | RLW1-54 | *crtW*2 | Astaxanthin |
| Library III** | TM3 | TM2 | TM2 | T7 | RLZ1-36 | *crtZ*2 | RLW1-54 | *crtW*2 | Astaxanthin |
| ZW1-12** | TM3 | TM2 | TM2 | T7 | RLZ | *crtZ*2 | RLW | *crtW*2 | Astaxanthin |
| ZW1** | TM3 | TM2 | TM2 | T7 | RLZ | *crtZ*2 | RLW | *crtW*2 | Astaxanthin |
| ZW2** | TM3 | TM2 | TM2 | T7 | RLZ | *crtZ*2 | RLW | *crtW*2 | Astaxanthin |
| ZW3 | TM3 | TM2 | TM2 | T7 | RLZ22 | *crtZ*2 | RLW16 | *crtW*2 | Astaxanthin |
| ZW4 | TM3 | TM2 | TM2 | T7 | RLZ30 | *crtZ*2 | RLW23 | *crtW*2 | Astaxanthin |
| ZW5 | TM3 | TM2 | TM2 | T7 | RLZ26 | *crtZ*2 | RLW33 | *crtW*2 | Astaxanthin |
| ZW6** | TM3 | TM2 | TM2 | T7 | RLZ | *crtZ*2 | RLW | *crtW*2 | Astaxanthin |
| ZW7** | TM3 | TM2 | TM2 | T7 | RLZ | *crtZ*2 | RLW | *crtW*2 | Astaxanthin |
| ZW8 | TM3 | TM2 | TM2 | T7 | RLZ28 | *crtZ*2 | RLW9 | *crtW*2 | Astaxanthin |
| ZW9** | TM3 | TM2 | TM2 | T7 | RLZ | *crtZ*2 | RLW | *crtW*2 | Astaxanthin |
| ZW10** | TM3 | TM2 | TM2 | T7 | RLZ | *crtZ*2 | RLW | *crtW*2 | Astaxanthin |
| ZW11 | TM3 | TM2 | TM2 | T7 | RLZ22 | *crtZ*2 | RLW50 | *crtW*2 | Astaxanthin |
| ZW12 | TM3 | TM2 | TM2 | T7 | RLZ22 | *crtZ*2 | RLW24 | *crtW*2 | Astaxanthin |
| ZW13-24** | TM3 | TM2 | TM2 | T7 | RLZ | *crtZ*2 | RLW | *crtW*2 | Astaxanthin |
| Z1 | TM3 | TM2 | TM2 | T7 | RLZ26 | *crtZ*2 | RW1 | *crtW*2 | Astaxanthin |
| Z2 | TM3 | TM2 | TM2 | T7 | RLZ25 | *crtZ*2 | RW1 | *crtW*2 | Astaxanthin |
| Z3 | TM3 | TM2 | TM2 | T7 | RLZ30 | *crtZ*2 | RW1 | *crtW*2 | Astaxanthin |
| Z4-6** | TM3 | TM2 | TM2 | T7 | RLZ | *crtZ*2 | RW1 | *crtW*2 | Astaxanthin |
| W1 | TM3 | TM2 | TM2 | T7 | RLZ22 | *crtZ*2 | RLW20 | *crtW*2 | Astaxanthin |
| W2 | TM3 | TM2 | TM2 | T7 | RLZ22 | *crtZ*2 | RLW27 | *crtW*2 | Astaxanthin |
| W3 | TM3 | TM2 | TM2 | T7 | RLZ22 | *crtZ*2 | RLW16 | *crtW*2 | Astaxanthin |
| W4-6** | TM3 | TM2 | TM2 | T7 | RLZ22 | *crtZ*2 | RLW | *crtW*2 | Astaxanthin |
| E,F** | TM3 | TM2 | TM2 | T7 | RLZ | *crtZ*2 | RLW | *crtW*2 | Astaxanthin |

* MG1655ΔABC - K12 MG1655 ΔrecAΔendAΔaroAΔaroBΔaroC with a T7 RNA polymerase (DE3) integrated. Promoter library – T7, TM1, TM2 and TM3 (Supplementary Table 7). RBS library – RZ1, RZ2, RZ3, RZ4, RW1, RW2, RW3 and RW4 (Supplementary Table 2), RLZ1-36 (Supplementary Table 3) and RLW1-54 (Supplementary Table 4). Gene library – crtY1 from Pantoea ananatis LMG20103 (Genbank no. ADD79327.1) and crtY2 from Uncultured marine bacterium HF10_19P19 (Genbank no. ABL60984.1). crtZ1 from Sulfolobus solfataricus P2 (Genbank no. AAK43015.1) and crtZ2 from Pantoea ananatis LMG20103 (Genbank no. ADD79330.1) and crtW1 from Anabaena variabilis ATCC 29413 (Genbank no. ABA23493.1) and crtW2 from Brevundimonas sp. SD212 (Genbank no. BAD99406.1).

****** The strains whose RBS sequence were unknown (they were either mixture of different strains or were not sequenced).

**Supplementary Table 2**. RBS sequences and predicted translation initiation rates for *crtZ* and *crtW*

| RBS name | Controlled Genes | Pre-Sequence + RBS sequence (Blue color) | Translation Initiation Rate (au) |
| --- | --- | --- | --- |
| RZ1 | ***crtZ1*** | CGGTCCCGGTACTGGCCGCTCTGCAGGCAATTATGACTACCCATCGCTAAGGTCGAACGAGATCACTACTA**AGGAAGGGATA** | 14790 |
| RZ2 | ***crtZ1*** | CGGTCCCGGTACTGGCCGCTCTGCAGGCAATTATGACTACCCATCGCTAAAGGTTCGATCGGAAACGATAATA**AGGGAGGTCGAA** | 113072 |
| RZ3 | ***crtZ2*** | CGGTCCCGGTACTGGCCGCTCTGCAGGCAATTATGACTACCCATCGCTAATTTTAAGTACCCCCTA**AGGAAGTCTCA** | 21389 |
| RZ4 | ***crtZ2*** | GGTCCCGGTACTGGCCGCTCTGCAGGCAATTATGACTACCCATCGCTAAAATA**AGGAGGTTAAAT** | 135699 |
| RW1 | ***crtW1*** | ATTCTGAAAGACGAATAGACTAAACAACACTAACACTAAAA**AGGGAGGTCAAA** | 78885 |
| RW2 | ***crtW1*** | ATTCTGAAAGACGAATAGACTAAACAACACTAACACTAAAAAGGGAGGTCAAACTCGCCTAACAGCGACGATATA**AGGAGGTTTATT** | 177766 |
| RW3 | ***crtW2*** | ATTCTGAAAGACGAATAGTTACCTACAATTACAAAAATA**AGGGGGTTCTTAC** | 40476 |
| RW4 | ***crtW2*** | ATTCTGAAAGACGAATAGTTACCTACAATTACAAAAATAAGGCACTAATTATAACCCATCACACTA**AGGAGGTAGCC** | 98792 |

**Supplementary Table 3.** RBS library for *crtZ*

| RBS name | RBS Sequence | Translation Initiation Rate (au) |
| --- | --- | --- |
| RLZ1 | CAGACTTGACGCAATTATAATAAGGAGGTTCAAAC | 83102 |
| RLZ2 | CAGACTTTACGCAATTATAATAAGGAGGTTCAAAC | 50654 |
| RLZ3 | CAAACTTGACGCAATTATAATAAGGAGGTTCAAAC | 43498 |
| RLZ4 | CAGACTTGACGCAATTATAATAAGGAGGATCAAAC | 40447 |
| RLZ5 | CAAACTTTACGCAATTATAATAAGGAGGTTCAAAC | 29805 |
| RLZ6 | CATACTTTACGCAATTATAATAAGGAGGATCAAAC | 29538 |
| RLZ7 | CAGACTTGACGCAATTATGATAAGGAGGTTCAAAC | 26976 |
| RLZ8 | CAGACTTTACGCAATTATAATAAGGAGGATCAAAC | 24654 |
| RLZ9 | CAAACTTGACGCAATTATAATAAGGAGGATCAAAC | 20240 |
| RLZ10 | CAAACTTTACGCAATTATAATAAGGAGGATCAAAC | 20058 |
| RLZ11 | CATACTTTACGCAATTATAATAAGGAGGTTCAAAC | 19701 |
| RLZ12 | CATACTTGACGCAATTATAATAAGGAGGTTCAAAC | 16801 |
| RLZ13 | CAGACTTTACGCAATTATGATAAGGAGGTTCAAAC | 16443 |
| RLZ14 | CAAACTTGACGCAATTATGATAAGGAGGTTCAAAC | 15039 |
| RLZ15 | CAGACTTTACGCAATTATAATAAGGAGGGTCAAAC | 12384 |
| RLZ16 | CAGACTTGACGCAATTATGATAAGGAGGATCAAAC | 7651 |
| RLZ17 | CATACTTGACGCAATTATAATAAGGAGGATCAAAC | 7473 |
| RLZ18 | CATACTTGACGCAATTATAATAAGGAGGATCAAAC | 6395 |
| RLZ19 | CAGACTTGACGCAATTATAATAAGGAGGGTCAAAC | 5867 |
| RLZ20 | CAGACTTTACGCAATTATGATAAGGAGGATCAAAC | 4664 |
| RLZ21 | CATACTTGACGCAATTATGATAAGGAGGTTCAAAC | 4355 |
| RLZ22 | CAGACTTTACGCAATTATGATAAGGAGGGTCAAAC | 4075 |
| RLZ23 | CAAACTTGACGCAATTATGATAAGGAGGATCAAAC | 3727 |
| RLZ24 | CATACTTTACGCAATTATGATAAGGAGGTTCAAAC | 3256 |
| RLZ25 | CAGACTTGACGCAATTATGATAAGGAGGGTCAAAC | 2973 |
| RLZ26 | CAAACTTTACGCAATTATAATAAGGAGGGTCAAAC | 2858 |
| RLZ27 | CATACTTTACGCAATTATAATAAGGAGGGTCAAAC | 2858 |
| RLZ28 | CAAACTTGACGCAATTATAATAAGGAGGGTCAAAC | 2085 |
| RLZ29 | CAAACTTGACGCAATTATGATAAGGAGGGTCAAAC | 1855 |
| RLZ30 | CAAACTTTACGCAATTATGATAAGGAGGGTCAAAC | 1855 |
| RLZ31 | CAAACTTTACGCAATTATGATAAGGAGGATCAAAC | 1585 |
| RLZ32 | CATACTTTACGCAATTATGATAAGGAGGGTCAAAC | 1542 |
| RLZ33 | CATACTTGACGCAATTATGATAAGGAGGATCAAAC | 1292 |
| RLZ34 | CATACTTTACGCAATTATGATAAGGAGGATCAAAC | 807 |
| RLZ35 | CATACTTGACGCAATTATGATAAGGAGGGTCAAAC | 585 |
| RLZ36 | CATACTTGACGCAATTATAATAAGGAGGGTCAAAC | 585 |

**Supplementary Table 4.** RBS library for *crtW*

| RBS name | RBS Sequence | Translation Initiation Rate (au) |
| --- | --- | --- |
| RLW1 | GCACGACCACCAGTTTAAGTTTAAGGAGGTATTA | 90288 |
| RLW2 | GCACGACCACCAGTTCAAGTTTAAGGAGGTATTA | 65889 |
| RLW3 | GCACGACCCCCAGTACAAGTTTAAGGAGGTATTA | 58545 |
| RLW4 | GCACGACCCCCAGTATAAGTTTAAGGAGGTATTA | 58545 |
| RLW5 | GCACGACCACCAGTGCAACTTTAAGGAGGTATTA | 43945 |
| RLW6 | GCACGACCCCCAGTTTAACTTTAAGGAGGTATTA | 40844 |
| RLW7 | GCACGACCCCCAGTTAAACTTTAAGGAGGTATTA | 37328 |
| RLW8 | GCACGACCCCCAGTTCAACTTTAAGGAGGTATTA | 34115 |
| RLW9 | GCACGACCCCCAGTAAAACTTTAAGGAGGTATTA | 32614 |
| RLW10 | GCACGACCCCCAGTATAACTTTAAGGAGGTATTA | 29806 |
| RLW11 | GCACGACCACCAGTGCAAGTTTAAGGAGGTATTA | 29309 |
| RLW12 | GCACGACCCCCAGTAAAAGTTTAAGGAGGTATTA | 28495 |
| RLW13 | GCACGACCCCCAGTTCAAGTTTAAGGAGGTATTA | 22753 |
| RLW14 | GCACGACCACCAGTGAAAGTTTAAGGAGGTATTA | 22373 |
| RLW15 | GCACGACCACCAGTTCAACTTTAAGGAGGTATTA | 22373 |
| RLW16 | GCACGACCACCAGTAAAAGTTTAAGGAGGTATTA | 17865 |
| RLW17 | GCACGACCCCCAGTGAAAGTTTAAGGAGGTATTA | 17369 |
| RLW18 | GCACGACCCCCAGTTTAAGTTTAAGGAGGTATTA | 15175 |
| RLW19 | GCACGACCCCCAGTGCAAGTTTAAGGAGGTATTA | 12675 |
| RLW20 | GCACGACCCCCAGTGAAACTTTAAGGAGGTATTA | 12117 |
| RLW21 | GCACGACCACCAGTGAAACTTTAAGGAGGTATTA | 10889 |
| RLW22 | GCACGACCACCAGTTAAACTTTAAGGAGGTATTA | 9095 |
| RLW23 | GCACGACCACCAGTTTAACTTTAAGGAGGTATTA | 9095 |
| RLW24 | GCACGACCCCCAGTACAACTTTAAGGAGGTATTA | 8843 |
| RLW25 | GCACGACCCCCAGTTAAAGTTTAAGGAGGTATTA | 8843 |
| RLW26 | GCACGACCCCCAGTGTAAGTTTAAGGAGGTATTA | 8082 |
| RLW27 | GCACGACCACCAGTAAAACTTTAAGGAGGTATTA | 7263 |
| RLW28 | GCACGACCACCAGTACAAGTTTAAGGAGGTATTA | 7263 |
| RLW29 | GCACGACCACCAGTATAAGTTTAAGGAGGTATTA | 7263 |
| RLW30 | GCACGACCACCAGTATAACTTTAAGGAGGTATTA | 7263 |
| RLW31 | GCACGACCACCAGTGTAAGTTTAAGGAGGTATTA | 7263 |
| RLW32 | GCACGACCCCCAGTGCAACTTTAAGGAGGTATTA | 6453 |
| RLW33 | GCACGACCCCCAGTGTAACTTTAAGGAGGTATTA | 5898 |
| RLW34 | GCACGACCACCAGTGTAACTTTAAGGAGGTATTA | 5300 |
| RLW35 | GCACGACCACCAGTTAAAGTTTAAGGAGGTATTA | 4232 |
| RLW36 | GCACGACCTCCAGTAAAAGTTTAAGGAGGTATTA | 3727 |
| RLW37 | GCACGACCTCCAGTAAAACTTTAAGGAGGTATTA | 3727 |
| RLW38 | GCACGACCTCCAGTACAAGTTTAAGGAGGTATTA | 3727 |
| RLW39 | GCACGACCTCCAGTACAACTTTAAGGAGGTATTA | 3727 |
| RLW40 | GCACGACCTCCAGTATAAGTTTAAGGAGGTATTA | 3727 |
| RLW41 | GCACGACCTCCAGTATAACTTTAAGGAGGTATTA | 3727 |
| RLW42 | GCACGACCTCCAGTGAAAGTTTAAGGAGGTATTA | 3727 |
| RLW43 | GCACGACCTCCAGTGAAACTTTAAGGAGGTATTA | 3727 |
| RLW44 | GCACGACCTCCAGTTAAAGTTTAAGGAGGTATTA | 3727 |
| RLW45 | GCACGACCTCCAGTTAAACTTTAAGGAGGTATTA | 3727 |
| RLW46 | GCACGACCTCCAGTTCAAGTTTAAGGAGGTATTA | 3727 |
| RLW47 | GCACGACCTCCAGTTCAACTTTAAGGAGGTATTA | 3727 |
| RLW48 | GCACGACCTCCAGTTTAAGTTTAAGGAGGTATTA | 3727 |
| RLW49 | GCACGACCTCCAGTTTAACTTTAAGGAGGTATTA | 3727 |
| RLW50 | GCACGACCACCAGTACAACTTTAAGGAGGTATTA | 2953 |
| RLW51 | GCACGACCTCCAGTGCAAGTTTAAGGAGGTATTA | 1734 |
| RLW52 | GCACGACCTCCAGTGCAACTTTAAGGAGGTATTA | 1734 |
| RLW53 | GCACGACCTCCAGTGTAAGTTTAAGGAGGTATTA | 1734 |
| RLW54 | GCACGACCTCCAGTGTAACTTTAAGGAGGTATTA | 1734 |

**Supplementary Table 5**. Astaxanthin production in engineered microbes

| No. | Hosts | Titer  (mg/L) | Content  (ppm) | Metabolic engineering strategies | References |
| --- | --- | --- | --- | --- | --- |
| 1 | *Saccharomyces cerevisiae* | / | 4700 | Combining the expression of *Haematococcus pluvialis* β-carotenoid hydroxylase (*crtZ*) and ketolase (*bkt*) genes with codon optimization, gene copy number adjustment, and iron cofactor supplementation. | 4 |
| 2 | *Saccharomyces cerevisiae* | / | 29 | Introduction of *E. uredovora* *CrtZ* and *CrtW* together with *BTS1, CrtI, CrtYB* | 5 |
| 3 | *Escherichia coli* | / | 2640 | Screening the combinatorial RBS library for *idi, crtE, crtB, crtI, lcyE, crtW* and *crtZ.* | 6 |
| 4 | *Escherichia coli* | / | 313 | Screening the best β-carotene hydroxylase genes | 7 |
| 5 | *Escherichia coli* | 2.07 | 1410 | Expression of *crtEBIY* along with the β-carotene-ketolase gene *crtW148* (NpF4798) and the β-carotene-hydroxylase gene (*crtZ*) | 8 |
| 6 | *Saccharomyces cerevisiae* | / | Detectable | Coexpression of *crtI, crtYB*, cytochrome P450 *crtS* and cytochrome P450 reductase *crtR* from *Xanthophyllomyces dendrorhous*. | 5 |
| 7 | *Escherichia coli* | 2.9 | 1993 | Explore the combination of 12 β-carotene ketolase and 4 β-carotene hydroxylase genes | 9 |
| 8 | *Corynebacterium glutamicum* | / | 1600 | Heterologous expression of *crtY*, β-carotene ketolase *crtW*, and hydroxylase *crtZ* and RBS engineering to balance *crtW* and *crtZ* | 10 |
| 9 | *Xanthophyllomyces dendrorhous* | 1.6 | 290 | Deletion of double CYP61 genes encoding C-22 sterol desaturases | 11 |
| 10 | *Xanthophyllomyces dendrorhous* | / | 9000 | Combined classical mutagenesis with genetic engineering of the complete pathway | 12 |
| 11 | *Saccharomyces cerevisiae* | 47.18 | 8100 | Enzyme engineering and directed evolution of the GGPP synthase and beta-carotene ketolase | 13 |
| 12 | *Kluyveromyces marxianus* | / | 9972 | Repeated genome integration of the key astaxanthin biosynthesis genes | 14 |
| 13 | *Escherichia coli* | 320 | 15,000 | Multidimensional Heuristic Process(MHP) and simultaneous fermentation and extraction | This study |

**Supplementary Table 6**. Degenerate oligos for cloning and RT-qPCR

| Degenerate oligos for cloning | |
| --- | --- |
| Oligo name | Sequence |
| I-T7P_lib-F | ATACGA*CTCACT*NNNRRGGAATTGTGAGCGGATA |
| I-T7P_lib-R | AGTGAG*TCGTAT*TAATTTCGCGGGATCG |
| I-RBSlib-*crtZ*2-F | GGAGGD*TCAAAC*ATGCTGTGGATCTGGAATG |
| I-RBSlib-*crtZ*2-R | GTTTGA*HCCTCC*TTATYATAATTGCGTMAAGTHTGTTAGCGATGGGTAGTCATA |
| RBSlib-*crtW*2-F | AAGGAG*GTATTA*ATGACCGCAGCTGTTGC |
| RBSlib-*crtW*2-R | TAATAC*CTCCTT*AAASTTDHACTGGDGGTCGTGCTTACTTACCAGATGCCGG |
|  | |
| Cloning primers | |
| I-pETend(-)-F | AAGGAAG*CTGAGTTG*GCTG |
| I-pETfro(-)-R | ATGTATATCT*CCTTCTTAA*AGTT |
| I-crtYpa(-)-R | TTAGCGATG*GGTAGTCATA*ATTG |
| I-crtYhf10(-)-R | TTACTTTTTG*TCAGCGTTG*TCG |
| I-crtYpa(pETr)-F | TTAAGAAGG*AGATATACAT*CGAGTAAGGAGGAGAACAATG |
| I-crtYpa(pETf)-R | CAACTCA*GCTTCCTT*TTAGCGATGGGTAGTCATAATTG |
| I-crtYhf10(pETr)-F | TTAAGAAGG*AGATATACAT*TGCATCCGTAAGTACAACATG |
| I-crtYhf10(pETf)-R | CAACTCA*GCTTCCTT*TTACTTTTTGTCAGCGTTGTCG |
| I-crtZss(Ypa)-F | TATGACTACC*CATCGCTAA*GGTCGAACGAGATCACTACTA |
| I-crtZss(Yhf10)-F | CAACGCTGA*CAAAAAGTAA*GGTCGAACGAGATCACTACTA |
| I-crtZss(pETf)-R | CAACTCA*GCTTCCTT*CTATTCGTCTTTCAGAATCGGA |
| I-crtZpa(Ypa)-F | TATGACTACC*CATCGCTAA*TTTTAAGTACCCCCTAAGGAAGTC |
| I-crtZpa(Yhf10)-F | CAACGCTGA*CAAAAAGTAA*TTTTAAGTACCCCCTAAGGAAGTC |
| I-crtZpa(pETf)-R | CAACTCA*GCTTCCTT*TTACTTACCAGATGCCGGTTC |
| I-crtWbs(Ypa)-F | TATGACTACC*CATCGCTAA*TTACCTACAATTACAAAAATAAGGG |
| I-crtWbs(Yhf10)-F | CAACGCTGA*CAAAAAGTAA*TTACCTACAATTACAAAAATAAGGG |
| I-crtWbs(pETf)-R | CAACTCA*GCTTCCTT*TCAGCTTTCGCCGCGCCA |
| I-crtWav(Ypa)-F | TATGACTACC*CATCGCTAA*ACTAAACAACACTAACACTAAAAAGGG |
| I-crtWav(Yhf10)-F | CAACGCTGA*CAAAAAGTAA*ACTAAACAACACTAACACTAAAAAGGG |
| I-crtWav(pETf)-R | CAACTCA*GCTTCCTT*TTAAAGGCTAATCTTGTATGCCTC |
| I-T7P(p15AR)-F | AGGCACC*GTGTATG*TCTTCCCCATCGGTGATG |
| I-bla(p15AF)-R | AAACAGA*AGCCACT*CTGACAGTTACCAATGCTTA |
| I-p15A(-)-F | AGTGGCT*TCTGTTT*CTATCA |
| I-p15A(-)-R | CATACAC*GGTGCCT*GACT |
|  |  |
| Oligos for quantitative reverse transcription PCR (RT-qPCR) | |
| rt-crtY1.Pa-F | TGGGCTTCCAGGCATTTA |
| rt-crtY1.Pa-R | AGACCGTGCGGGTGAGAC |
| rt-crtZ1-F-163 | AACAGGCAGAACTGGAAA |
| rt-crtZ1-R-218 | GGATACGGATGCGAACA |
| rt-crtZ2-F | AATCGGCATGGAAGTGGT |
| rt-crtZ2-R | GGTTCGTGATGGGACAGG |
| rt-crtW1-F | TTTTCTGTGGGCAATCTC |
| rt-crtW1-R | CTGGTTGATGATGGAGGTA |
| rt-crtW2-F | CGCACGTATTTCGGATGG |
| rt-crtW2-R | TCAGCAGGTTGGCAGGAC |
| rt-cysG-F | TTGTCGGCGGTGGTGATGTC |
| rt-cysG-R | ATGCGGTGAACTGTGGAATAAACG |

‘*’ in the sequence represents a phosphorothioate bond.

**Supplementary Table 7**. Characterization of the promoter library

| Promoter | Sequence | Transcription efficiency |
| --- | --- | --- |
| T7 | TAATACGACTCACTATAGGGGA | 100% |
| TM1 | TAATACGACTCACTAATGGGGA | 92% |
| TM2 | TAATACGACTCACTCGAGGGGA | 37% |
| TM3 | TAATACGACTCACTATAAAGGA | 16% |

# Supplementary References

1. Farasat, I. et al. Efficient search, mapping, and optimization of multi-protein genetic systems in diverse bacteria. *Mol. Syst. Biol.* **10**, 731-731 (2014).

2. Bonde, M.T. et al. Predictable tuning of protein expression in bacteria. *Nat. Methods* **13**, 233-236 (2016).

3. Kujawski, J., Bernard, M.K., Janusz, A. & Kuźma, W. Prediction of log P: ALOGPS Application in Medicinal Chemistry Education. *J. Chem. Educ.* **89**, 64-67 (2012).

4. Zhou, P., Ye, L., Xie, W., Lv, X. & Yu, H. Highly efficient biosynthesis of astaxanthin in Saccharomyces cerevisiae by integration and tuning of algal crtZ and bkt. *Appl. Microbiol. Biotechnol.* **99**, 8419-8428 (2015).

5. Ukibe, K., Hashida, K., Yoshida, N. & Takagi, H. Metabolic engineering of Saccharomyces cerevisiae for astaxanthin production and oxidative stress tolerance. *Appl. Environ. Microbiol.* **75**, 7205-7211 (2009).

6. Zelcbuch, L. et al. Spanning high-dimensional expression space using ribosome-binding site combinatorics. *Nucleic Acids Res.* **41**, e98 (2013).

7. Scaife, M.A., Ma, C.A., Ninlayarn, T., Wright, P.C. & Armenta, R.E. Comparative Analysis of β-Carotene Hydroxylase Genes for Astaxanthin Biosynthesis. *J. Nat. Prod.* **75**, 1117-1124 (2012).

8. Lemuth, K., Steuer, K. & Albermann, C. Engineering of a plasmid-free Escherichia coli strain for improved in vivo biosynthesis of astaxanthin. *Microb. Cell. Fact.* **10**, 29 (2011).

9. Scaife, M.A., Burja, A.M. & Wright, P.C. Characterization of cyanobacterial beta-carotene ketolase and hydroxylase genes in Escherichia coli, and their application for astaxanthin biosynthesis. *Biotechnol. Bioeng.* **103**, 944-955 (2009).

10. Henke, N.A., Heider, S.A., Peters-Wendisch, P. & Wendisch, V.F. Production of the Marine Carotenoid Astaxanthin by Metabolically Engineered Corynebacterium glutamicum. *Mar. Drugs* **14** (2016).

11. Yamamoto, K. et al. Enhancement of astaxanthin production in Xanthophyllomyces dendrorhous by efficient method for the complete deletion of genes. *Microb. Cell. Fact.* **15**, 155 (2016).

12. Gassel, S., Breitenbach, J. & Sandmann, G. Genetic engineering of the complete carotenoid pathway towards enhanced astaxanthin formation in Xanthophyllomyces dendrorhous starting from a high-yield mutant. *Appl. Microbiol. Biotechnol.* **98**, 345-350 (2014).

13. Zhou, P. et al. Alleviation of metabolic bottleneck by combinatorial engineering enhanced astaxanthin synthesis in Saccharomyces cerevisiae. *Enzyme Microb. Technol.* **100**, 28-36 (2017).

14. Lin, Y.-J. et al. Metabolic Engineering a Yeast to Produce Astaxanthin. *Bioresour. Technol.* (2017).
